# Evaluation of drug administration errors in a teaching hospital

**DOI:** 10.1186/1472-6963-12-60

**Published:** 2012-03-12

**Authors:** Sarah Berdot, Brigitte Sabatier, Florence Gillaizeau, Thibaut Caruba, Patrice Prognon, Pierre Durieux

**Affiliations:** 1 Department of pharmacy, Hôpital européen Georges Pompidou, Assistance Publique - Hôpitaux de Paris, F-75015 Paris, France; 2 INSERM, UMR S 872, Equipe 20, Centre de Recherche des Cordeliers, F-75006 Paris, France; 3 INSERM, U765, F-75006 Paris, France; 4 INSERM, Centre D'investigation Épidémiologique 4, F-75015 Paris, France; 5 Laboratoire Interdisciplinaire de Recherche en Economie de Santé, EA4410, Université Paris Descartes, Sorbonne Paris Cité, F-75006 Paris, France; 6 Université Paris-Sud 11, 92290 Chatenay-Malabry, France; 7 Université Paris Descartes, Sorbonne Paris Cité, Faculté de médecine, F-75006 Paris, France; 8 Department of Hospital Informatics, Hôpital européen Georges Pompidou, Assistance Publique - Hôpitaux de Paris, 20 rue Leblanc, F-75015 Paris, France

**Keywords:** Hospital care, Medication errors, Direct observation

## Abstract

**Background:**

Medication errors can occur at any of the three steps of the medication use process: prescribing, dispensing and administration. We aimed to determine the incidence, type and clinical importance of drug administration errors and to identify risk factors.

**Methods:**

Prospective study based on disguised observation technique in four wards in a teaching hospital in Paris, France (800 beds). A pharmacist accompanied nurses and witnessed the preparation and administration of drugs to all patients during the three drug rounds on each of six days per ward. Main outcomes were number, type and clinical importance of errors and associated risk factors. Drug administration error rate was calculated with and without wrong time errors. Relationship between the occurrence of errors and potential risk factors were investigated using logistic regression models with random effects.

**Results:**

Twenty-eight nurses caring for 108 patients were observed. Among 1501 opportunities for error, 415 administrations (430 errors) with one or more errors were detected (27.6%). There were 312 wrong time errors, ten simultaneously with another type of error, resulting in an error rate without wrong time error of 7.5% (113/1501). The most frequently administered drugs were the cardiovascular drugs (425/1501, 28.3%). The highest risks of error in a drug administration were for dermatological drugs. No potentially life-threatening errors were witnessed and 6% of errors were classified as having a serious or significant impact on patients (mainly omission). In multivariate analysis, the occurrence of errors was associated with drug administration route, drug classification (ATC) and the number of patient under the nurse's care.

**Conclusion:**

Medication administration errors are frequent. The identification of its determinants helps to undertake designed interventions.

## Background

Since the release of the report *To Err Is Human*, patient safety has risen to the forefront of healthcare issues [1]. Medication errors can occur during any of the three steps of the medication use process: from prescription, medication delivery to dispensing to the patient. Reviews on medication errors [2-10], prescription errors [11] or dispensing errors [12] are numerous. The evaluation and improvement of drug administration process are key elements in patient safety. A review of drug administration errors detected by the observation technique [13] revealed methodological limitations in studies evaluating the administration process: no standardized definition of error types and error rate, lack of information about the selection method of nurses observed and number of nurses observed, the level of experience of nurses, the number of patients or information on the observation technique [14-28].

To overcome the limits described above, we aimed to assess the frequency, type, potential clinical significance and determinants of drug administration errors detected by direct observation in adult in-patients.

## Methods

### Setting

The study was conducted in four adult wards (90 beds) at a teaching hospital in Paris, France (800 beds): immunology-cardiology ward, nephrology ward, vascular medical ward and cardiovascular surgical ward. These four wards are the only ones for which the drug doses are prepared daily by the pharmacy. Prescriptions were written by physicians, using the same computerized physician order entry system (DxCare^®^, Medasys). The hospital pharmacy validated orders and delivered the prescribed drugs to each ward, on a unit-dose basis except for drugs prescribed as needed. If necessary, the nurses obtained the drugs from secure and automated medication cabinets (Omnicell^® ^Inc.) available in each ward. Each nurse was in charge of 6 to 8 patients.

### Study design

Disguised direct observation was used for the detection of drug administration errors [13], because this approach gives more efficient, objective, and reliable results than spontaneous reporting or patient chart reviews [29-31]. Head nurses and physicians were informed about the objectives and the nurses who were observed were told that a pharmacist was evaluating the process of drug administration with the aim of improving it. We used a single observer, to avoid problems of interobserver variability. At the start of the observation period, each nurse gave written consent for observation and had the option of refusing to be observed. The observer was a clinical pharmacist and received a one-month training with a senior pharmacist before the start of the study.

For each observation round and ward, the observation order of nurses was randomized for identification of the nurse to be observed. Patients were not selected. The observer watched the selected nurses preparing and administering medication. Observations were carried out on six consecutive days per ward (including Saturday), for three drug rounds per day (8 am, 12 pm, 6 pm). Recorded observations were compared immediately after observation with the physician orders. Because the observer saw the order after drug administration, she was unable to prevent some errors. But if she was aware of an imminent potential error, she intervened to prevent it.

Emergency drugs, parenteral nutrition and drugs prescribed as needed were not observed as the delivery of these medications did not follow the unit-dose process described above. Non-permanent nurses were not included in the study.

The following data were collected: characteristics of the nurse (age, sex, years of experience and years in the unit); nurse-to-patient ratio; nurse workload (number of patients under the care of each nurse, including patients who were admitted on that day) and number of interruptions during the drug administration round, and characteristics of the drugs (Anatomical Therapeutic Chemical (ATC) classification, unit-dose prepared by the pharmacy, route, pharmaceutical form, dose and time of drug administration).

The study was not considered as research but as routine care. It was part of an audit for quality improvement and was considered exempted from ethical approval. All data were studied anonymously.

### Outcomes

The main outcome assessed was error rate. Error rate without wrong time errors, types of errors, severity of errors and risk factors were also evaluated.

### Errors

To overcome the limits found in the review we conducted, we standardized the drug administration error rate using the same denominator (see below) and the types of errors defined by the American Society of Health-system Pharmacists (ASHP). Drug administration errors were classified into the nine categories of the ASHP: omission error (failure to administer an ordered dose to a patient), wrong time error (administered more than one hour before or after the specified time), unauthorized drug error (dose given to the wrong patient, unordered drugs), wrong dose error, wrong dosage-form error, wrong drug-preparation error (incorrect dilution or reconstitution, mixing drugs that are physically incompatible and inadequate product packaging), wrong administration technique error (doses administered via the wrong route (different from the route prescribed), via the correct route but at the wrong site, and at the wrong rate of administration), deteriorated drug error (use of expired drugs or improperly stored drugs) and other medication error (included any drug administration errors not fitting into the above predefined categories) [32]. The error rate was calculated using the Total Opportunities for Errors (TOE), which is the sum of all doses ordered plus all the unordered doses given [33]. The drug administration error rate was then calculated as the number of administrations with one or more errors divided by the TOE and multiplied by 100. We also calculated the error rate without wrong time errors, as this type of error is a matter of much debate. Some authors recommended that studies on medication errors should report both the error rates with and without timing errors [29,34]. For injectable drugs, an administration of drug and solvent corresponded to one opportunity for error. Any given administration could be subject to several types of error, so it was possible that the sum of errors types was greater than the total number of administrations with at least one error. But an error could only be classified in one category of error.

A panel of senior experts composed of four physicians, three head nurses and two pharmacists evaluated the severity of each error anonymously according to a three-category scale: no clinical impact, serious or significant clinical impact, life-threatening impact on the patient [35].

### Statistical analysis

Categorical variables are reported as frequencies (percentages) and numerical variables are reported as medians (minimum and maximum). The drug administration error rate was calculated with and without wrong time errors. We investigated the relationship between the occurrence of errors (error rate and error rate without wrong time errors) and potential risk factors (characteristics of the nurse and drug), using logistic regression models with random effects (intercepts) to take multiple observations for the same patient and the same nurse into account. All risk factors were analyzed in univariable and multivariable analyses. The final model was obtained by removing all factors not significant at the 5% level. Results are expressed as odds ratios (OR), with the 95% confidence intervals (CI). Data were analyzed with SAS^® ^version 9.1 (SAS Institute, Cary, NC, USA).

## Results

### Study participants

Disguised direct observation was carried out during 72 drug rounds. All the nurses agreed to participate. Table 1 lists the characteristics of the nurses. There were 28 nurses (all female): six in the immunology-cardiology ward, seven each in the nephrology ward and cardiovascular surgical ward and eight in the vascular medical ward. Half the nurses were observed at least twice (min-max: 1-6 observation periods). Drug rounds lasted a median of 1 h 12 min (min-max: 15 min-2 h 45 min). During the study period, 108 patients were under the care of the nurses studied. A median of six patients per drug round was observed (min-max: 2-9).

**Table 1:** Characteristics of the nurses

Nurses	N = 28
Age, median [min - max]	29 [21-50]
Women, n (%)	28 (100)
Years of experience, median [min - max]	5 [0.8-27.5]
Years in the unit, median [min - max]	3.3 [0.03-10.0]
Full-time job, n (%)	26 (93)

### Error rate

In total, we recorded 1501 TOE. At least one error was detected in 415 of the 1501 TOE (error rate = 27.6%), with 430 errors identified in total. For 13 administrations, 2 errors were observed and for one administration, 3 errors were observed. Of the 14 administrations with more than one error, 10 included a wrong time error (see details in footnotes of Table 2). After exclusion of the 312 wrong time errors, 113 of the 1501 administrations (TOE) remained erroneous (415-312 + 10) corresponding to an error rate without wrong time error of 7.5%.

**Table 2:** Types of drug administration errors related to drug ATC classification

Drug ATC*	Number of TOE (column%)	Number of errors (column%)	Number of TOE† with errors (column%)	Error rate‡
A	270 (18.0)	90 (20.9)	86 (20.7)^a^	31.8%
B	201 (13.4)	38 (9.5)	38 (9.2)	18.9%
C	425 (28.3)	89 (20.7)	87 (21.0)^b^	20.5%
D	8 (0.5)	7 (1.7)	7 (1.7)	87.5%
G	26 (1.7)	6 (1.5)	6 (1.4)	23.1%
H	40 (2.7)	18 (4.2)	16 (3.9)^c^	40.0%
J	150 (10.0)	55 (12.8)	50 (12.0)^d^	33.3%
L	22 (1.5)	3 (0.7)	3 (0.7)	13.6%
M	13 (0.9)	1 (0.2)	1 (0.2)	7.7%
N	279 (18.6)	96 (22.3)	94 (22.7)^e^	33.7%
R	36 (2.4)	15 (3.7)	15 (3.6)	41.7%
S	10 (0.7)	6 (1.5)	6 (1.4)	60.0%
V	16 (1.1)	4 (1.0)	4 (1.0)	25.0%
Others	5 (0.3)	2 (0.5)	2 (0.5)	40.0%
All	**1501**	**430**	**415**	**27.6%**

* ATC classification detailed in additional file

† TOE: Total Opportunities for Errors

‡ Error rate = Number of TOE with errors/Number of TOE

^a ^4 administrations with two types of errors (Wrong time error + Wrong drug-preparation error: 3, Wrong drug-preparation error + Wrong administration technique error: 1)

^b ^2 administrations with two types of errors (Wrong time error + Unauthorized drug error: 1, Wrong time error + Wrong administration technique error: 1)

^c ^2 administrations with two types of errors (Wrong time error + Other medication error: 1, Wrong dose error + Other medication error: 1)

^d ^3 administrations with two types of errors (Wrong time error + Wrong dosage-form error: 1, Wrong dose error + Wrong drug-preparation error: 1, Wrong drug-preparation error + Wrong administration technique error: 1), 1 administration with three types of errors (Wrong time error + Unauthorized drug error + Wrong drug-preparation error)

^e ^2 administrations with two types of errors (Wrong time error + Wrong dose error: 1, Wrong time error + Wrong dosage-form error: 1)

### Types of errors

Wrong time errors were the principal type of errors observed (*n *= 312, 72.6%), followed by errors of omission (*n *= 60, 14.0%), and unauthorized drug errors (n = 16, 3.7%). There were 10 errors (2.3%) with the type "Other medication error" corresponding to administration of thyroid hormones with food, whereas these drugs should be administered to fasting patients. Wrong dose errors, wrong dosage-form errors, wrong drug-preparation errors, and wrong administration technique errors were rare (n = 8 for each type, 1.9%). There was no deteriorated drug error (see additional file 1).

### Drug classification

The type of drugs administered and the error rate according to ATC drug classification are described in Table 2 and Additional file 1. The most frequently administered drugs were the cardiovascular drugs (425/1501, 28.3%) (C in ATC classification), the central nervous system drugs (279/1501, 18.6%) (N in ATC classification), followed by the gastrointestinal drugs (270/1501, 18.0%) (A in ATC classification).

More than a half of administrations of dermatological drugs (D in ATC classification) and sensory organs drugs (S in ATC classification) had an error but they were rarely prescribed (0.5% and 0.7% respectively). The most frequently administered drugs (C, N and A in ATC classification) had error rates of 20.5%, 33.7% and 31.8% respectively.

The administration or omission of 182 different drugs were incorrect. The first ten most administered drugs were oral acetaminophen (37% of administrations had an error), esomeprazol (37%), acetylsalicylic acid (22%), oral furosemide (9%), bisoprolol (28%), atorvastatin (9%), calcium heparin (32%), oral tramadol (22%), amlodipine (16%) and oral potassium chloride (31%).

### Severity of errors

The observer intervened three times to prevent errors from occurring: corresponding to 5 drugs almost administered to the wrong patient, one ganciclovir administered at the wrong dose (100 mg instead of 300 mg) and one amoxicillin/clavulanic acid drug almost administered instead of prescribed amoxicillin. No patient harm was observed.

The expert panel classified 406 (94%) of the 430 errors as having no clinical impact on the patient and 24 (6%) as having serious or significant impact. Most of these errors were omissions. No potentially life-threatening errors were witnessed.

### Risk factors for errors

Univariable logistic regression analysis indicated that the occurrence of errors was significantly associated with administration route, drug ATC and number of patients under the nurse's care (Table 3). There was a non significant trend towards an increase in the occurrence of errors towards the end of the week. There was a non significant trend towards a decrease in the occurrence of errors with increase age of the nurse (OR associated with a five-year increase in age: 0.81 [95% CI: 0.62 to 1.06], *p *= 0.127). No significant effects of clinical unit, week day, drug round, unit-dose preparation by the pharmacy, interruptions and number of patients receiving drugs were found. The removal of medication from secure medication cabinets, by the nurses, was not associated with errors.

**Table 3:** Association between the occurrence of errors and general factors

	Administrations		Univariable analysis		Multivariable analysis*	
Variable, n (%)	No error (N = 1086)	Error (N = 415)	**OR [95% CI]**†	**Global P**†	**OR [95% CI]**†	**Global P**†
**Unit**				0.64		
*Nephrology*	230 (21.2)	54 (13.0)	1			
*Immunology-Cardiology*	309 (28.5)	125 (30.1)	1.29 [0.37-4.52]			
*Vascular medical*	266 (24.5)	113 (27.2)	1.56 [0.48-5.15]			
*Cardiovascular surgical*	281 (25.9)	123 (29.6)	2.17 [0.65-7.23]			
**Day**				0.104		
*Tuesday*	192 (17.7)	56 (13.5)	1			
*Monday*	175 (16.1)	51 (12.3)	1.52 [0.88-2.64]			
*Wednesday*	199 (18.3)	73 (17.6)	1.43 [0.75-2.70]			
*Thursday*	168 (15.5)	95 (22.9)	2.56 [1.33-4.92]			
*Friday*	165 (15.2)	86 (20.7)	1.81 [0.95-3.45]			
*Saturday*	187 (17.2)	54 (13.0)	1.69 [0.80-3.56]			
**Drug round**				0.68		
*Noon*	140 (12.9)	61 (14.7)	1			
*Morning*	504 (46.4)	169 (40.7)	0.87 [0.59-1.29]			
*Night*	442 (40.7)	185 (44.6)	0.77 [0.40-1.46]			
**Route**				< .0001		< .0001
*Oral*	975 (89.8)	327 (78.8)	1		1	
*Injectable*	81 (7.5)	50 (12.0)	2.42 [1.58-3.71]		3.30 [2.01-5.44]	
*Other*	30 (2.8)	30 (7.2)	4.61 [2.39-8.89]		1.89 [0.72-4.94]	
Drug ATC**				< .0001		< .0001
*C*	338 (31.1)	87 (21.0)	1		1	
*M*	12 (1.1)	1 (0.2)	0.28 [0.03-2.32]		0.30 [0.04-2.53]	
*L*	19 (1.7)	3 (0.7)	0.51 [0.13-1.93]		0.65 [0.17-2.48]	
*B*	163 (15.0)	38 (9.2)	0.95 [0.59-1.53]		0.61 [0.36-1.03]	
*V*	12 (1.1)	4 (1.0)	1.03 [0.25-4.25]		1.09 [0.26-4.54]	
*G*	20 (1.8)	6 (1.4)	1.51 [0.52-4.42]		1.51 [0.51-4.42]	
*Others*	3 (0.3)	2 (0.5)	1.60 [0.20-12.65]		1.47 [0.18-11.69]	
*A*	184 (16.9)	86 (20.7)	1.69 [1.12-2.53]		1.61 [1.06-2.42]	
*N*	185 (17.0)	94 (22.7)	1.94 [1.30-2.91]		2.01 [1.34-3.02]	
*J*	100 (9.2)	50 (12.0)	2.61 [1.58-4.30]		1.96 [1.16-3.30]	
*H*	24 (2.2)	16 (.3.9)	3.38 [1.58-7.26]		3.35 [1.57-7.18]	
*R*	21 (1.9)	15 (3.6)	4.49 [1.86-10.85]		3.09 [1.06-8.99]	
*S*	4 (0.4)	6 (1.4)	7.55 [1.51-37.74]		4.49 [0.68-29.66]	
*D*	1 (0.1)	7 (1.7)	38.28 [3.85-380.34]		26.10 [2.17-314.30]	
**Unit-dose prepared by the pharmacy**				0.22		
No	216 (19.9)	98 (23.6)	1			
Yes	870 (80.1)	317 (76.4)	0.81 [0.58-1.13]			
**Interruptions**††				0.82		
No	1037 (95.5)	339 (95.8)	1			
Yes	49 (4.5)	15 (4.2)	0.92 [0.47-1.82]			
**Nurse's age**‡‡	30 (27-35)	29 (27-33)	0.81 [0.62-1.06] ‡	0.127		
**Number of patient under nurse's care**‡‡	8 (7-9)	9 (7-9)	1.21 [1.05-1.41] #	0.011	1.22 [1.04-1.42] #	0.013
**Number of patient with drugs to be administered‡‡**	6 (6-8)	7 (6-8)	1.04 [0.92-1.18] #	0.50		

* Terms not significant at the 5% level were removed (backward selection)

† Derived from the logistic regression model with random effects

‡ OR for a 5-year increase

# OR for a 1-patient increase

** ATC classification detailed in additional file

†† Errors were observed on 354 administrations only (and not 415) due to omissions.

‡‡ Median (Q1-Q3)

In the multivariable analysis (Table 3), the same three factors were associated with the occurrence of errors. The risk of error was higher for administrations by injection (OR versus the oral route: 3.30 [95% CI: 2.01 to 5.44], *p *< 0.001) and for nurses with larger numbers of patients under their care (OR associated with a one-patient increase: 1.22 [95% CI: 1.04 to 1.42], *p *= 0.013). The highest risks of error in a drug administration were for dermatological drugs (D in ATC classification) and sensory organs drugs (S in ATC classification) but the confidence intervals were large due to small sizes. The risk of error was significantly higher for respiratory system drugs (R in ATC classification), systematic hormonal drugs (H in ATC classification), anti-infective drugs for systemic use (J in ATC classification), central nervous system drugs (N in ATC classification) and gastrointestinal drugs (A in ATC classification) than for cardiovascular drugs, the most frequently administered drugs (C in ATC classification). Analysis of risk factors for errors excluding wrong time errors highlighted the same factors. Since the frequency of event was more rare (113/1501), the drug ATC (14 levels) could not be evaluated as a risk factor in a model with random effects. Drug ATC was highly significant in the univariate analysis (logistic regression model without random effects: *p *< 0.001). The multivariable analysis (without drug ATC) indicated that the risk of error (wrong time errors excluded) was higher for administrations by injection (OR versus the oral route: 6.89 [95% CI: 4.06 to 11.70], *p *< 0.001), tended to be lower with increase age of the nurse (OR associated with a five-year increase in age: 0.78 [95% CI: 0.60 to 1.02], *p *= 0.069), and surprisingly there was a non significant trend towards a decrease of risk of error (wrong time errors excluded) with an increase of the number of patients under the nurse's care (OR associated with a one-patient increase: 0.75 [95% CI: 0.56 to 1.01], *p *= 0.060).

## Discussion

Drug administration errors were common in the wards studied. An error rate of 27.6% was found, decreasing to 7.5% when wrong time errors were excluded. Most of the errors (94%) were unlikely to cause lasting harm, but 6% were serious. By extrapolation to the whole hospital (800 beds), a rate of 6% serious errors would have meant more than 200 such errors every month. The factors associated with errors were administration route, drug ATC and number of patients under the nurse's care. Unit-dose preparation by the pharmacy was not associated with a higher occurrence of errors than the removal of the drug from a secure medication cabinet.

In the literature, there is a high heterogeneity in the methodologies, leading to heterogeneity in the results. In the systematic review we conducted, wrong time errors followed by omissions are the most frequent types of errors reported. In addition, the error rate without wrong time errors ranges from 1% [36,37] to 48% [38]. In a previous study in two adult units (geriatric and cardiovascular-thoracic surgery unit), error rates reached 14.9% with wrong time errors and 11% without wrong time errors [39]. The total error rate in our study was higher than those in the study by Chua and colleagues (11.4% in an adult hematology unit), but the error rates without wrong time errors was similar to that reported here (8.7%) [18].

We did not find an association with the unit, the day of observation and the drug rounds. As our study, Prot and colleagues found an association between errors and drug ATC classification (cardiovascular, anti-infective and central nervous system drugs) [23]. Chua and colleagues showed an association between errors and injectable route administration compared to oral route [18]. Finally, nurse workload was a risk factor of medication administration errors in the study by Tissot and colleagues whereas injectable administration was not associated with errors [39].

We chose to use disguised observation technique in this study. The observation period was relatively long. We used a single observer specifically trained for this study. To overcome the limits found in our review, we standardized the drug administration error rate using the same denominator (TOE) and the types of errors (ASHP classification). We reported the number of drug administration's with one or more errors in order to calculate an error rate excluding some types of errors. Finally, characteristics of hospital are presented (country, types of units, delivery system, characteristics of nurses observed). Our study has several potential limitations. First, it was a single-center study. Observation is very time-consuming and can therefore be carried out for very long periods of time. We did not observe nurses during the Sundays, and thus the applicability of the results for work at these times is unknown. It is also possible that nurses changed their behaviors when observed because they were aware that they were being observed to identify problems in the medication use process. However, Allan and Barker showed that disguised observation decreases the Hawthorne effect on observed nurses [29]. We did not observe non-permanent nurses as their agreement could not be obtain. However they represented less than 10% of the nurses during the observation period. Medication with high risk like chemotherapy drugs were rarely prescribed in the 4 units observed therefore no error of such administration was detected.

Different interventions have been proposed to improve the drug administration process. This study shows that those interventions should be adapted to the local context and the type of errors observed. For example, introduction of bar-code medication administration systems together with awareness of nurses could reduce some errors like omissions and wrong time (the two most frequent errors found in our study). Wrong administration technique including injectable drugs could be decrease with nurse training and awareness to manipulate injectable drugs.

## Conclusion

Medication safety issues are an important element of the medication use process in hospitals. Drug administration errors are frequent. Standardization of drug administration error rate using the same denominator (TOE) and types of errors remains essential for further studies.

## Competing interests

All authors declared that they have no competing interests: no support from any organization for the submitted work; no financial relationships with any organization that might have an interest in the submitted work in the previous five years; no other relationships or activities that could appear to have influenced the submitted work. SB as PhD student was funded by HMR Expert and a grant from AP-HP (from November 2010 to October 2011) but none of these funds has influenced the submitted work.

## Authors' contributions

SB, BS, FG, TC, PP and PD conceived and planned the work that led to the manuscript. SB made substantial contributions in the acquisition, analysis and interpretation of the data. FG performed data analysis made substantial contributions in the interpretation of the data. BS and PD made substantial contributions in the interpretation of the data.

SB wrote the first draft of the report. All authors were involved in drafting and revising the various drafts of the report with substantive suggestions. All authors read and approved the final manuscript.

## Pre-publication history

The pre-publication history for this paper can be accessed here:

http://www.biomedcentral.com/1472-6963/12/60/prepub

## Supplementary Material

Additional file 1 **Types of drug administration errors related to drug ATC classification**. Click here for file
