# Supplementary material for: Evaluation of drug administration errors in a teaching hospital
Source: BMC Health Serv Res. 2012 Mar 12;12:60. doi: 10.1186/1472-6963-12-60 (PMC3364158; doi:10.1186/1472-6963-12-60)
Supplement: Additional file 1 — Types of drug administration errors related to drug ATC classification. [file 1472-6963-12-60-S1.DOC]

Table Additional file: Types of drug administration errors related to drug ATC classification

| Types of errors | Omission error | Wrong time error | Unauthorized drug error | Wrong dose error | Wrong dosage-form error | Wrong drug-preparation error | Wrong administration technique error | Deteriorated drug error | Other medication error |
| --- | --- | --- | --- | --- | --- | --- | --- | --- | --- |
| Drug ATC* | n (%) | n (%) | n (%) | n (%) | n (%) | n (%) | n (%) | n (%) | n (%) |
| A | 6 (10.0) | 69 (22.1) | 4 (25.0) | 1 (12.5) | 1 (12.5) | 4 (50.0) | 5 (62.5) |  |  |
| B | 6 (10.0) | 30 (9.6) | 2 (12.5) |  |  |  |  |  |  |
| C | 1 (1.7) | 82 (26.3) | 4 (25.0) |  |  |  | 2 (25.0) |  |  |
| D | 6 (10.0) | 1 (0.3) |  |  |  |  |  |  |  |
| G |  | 6 (1.9) |  |  |  |  |  |  |  |
| H |  | 5 (1.6) |  | 2 (25.0) |  | 1 (12.5) |  |  | 10 (100.0) |
| J | 8 (13.3) | 35 (11.2) | 4 (25.0) | 2 (25.0) | 2 (25.0) | 3 (37.5) | 1 (12.5) |  |  |
| L |  | 3 (1.0) |  |  |  |  |  |  |  |
| M |  | 1 (0.3) |  |  |  |  |  |  |  |
| N | 24 (40.0) | 63 (20.2) | 1 (6.3) | 3 (37.5) | 5 (62.5) |  |  |  |  |
| R | 6 (10.0) | 9 (2.9) |  |  |  |  |  |  |  |
| S | 2 (3.3) | 4 (1.3) |  |  |  |  |  |  |  |
| V | 1 (1.7) | 2 (0.6) | 1 (6.3) |  |  |  |  |  |  |
| Others |  | 2 (0.6) |  |  |  |  |  |  |  |
| All | **60 (100)** | **312 (100)** | **16 (100)** | **8 (100)** | **8 (100)** | **8 (100)** | **8 (100)** | **0** | **10 (100)** |

*A (alimentary tract and metabolism), B (blood and blood-forming organs), C (cardiovascular system), D (dermatological), G (genitourinary system and sex hormones), H (systematic hormonal preparation), J (anti-infective drugs for systemic use), L (antineoplastic and immunomodulating agents), M (musculoskeletal system), N (nervous system), R (respiratory system), S (sensory organs), V (various), Others (without ATC)

† OE : Opportunity for Errors, TOE : Total Opportunities for Errors
